# T-cell metabolism in autoimmune disease

**DOI:** 10.1186/s13075-015-0542-4

**Published:** 2015-02-11

**Authors:** Zhen Yang, Eric L Matteson, Jörg J Goronzy, Cornelia M Weyand

**Affiliations:** Department of Medicine, Stanford University School of Medicine, CCSR Building Rm 2225, 269 Campus Drive West, Stanford, CA 94305-5166 USA; Division of Rheumatology, Mayo Clinic College of Medicine, Rochester, MN 55905 USA

## Abstract

Cancer cells have long been known to fuel their pathogenic growth habits by sustaining a high glycolytic flux, first described almost 90 years ago as the so-called Warburg effect. Immune cells utilize a similar strategy to generate the energy carriers and metabolic intermediates they need to produce biomass and inflammatory mediators. Resting lymphocytes generate energy through oxidative phosphorylation and breakdown of fatty acids, and upon activation rapidly switch to aerobic glycolysis and low tricarboxylic acid flux. T cells in patients with rheumatoid arthritis (RA) and systemic lupus erythematosus (SLE) have a disease-specific metabolic signature that may explain, at least in part, why they are dysfunctional. RA T cells are characterized by low adenosine triphosphate and lactate levels and increased availability of the cellular reductant NADPH. This anti-Warburg effect results from insufficient activity of the glycolytic enzyme phosphofructokinase and differentiates the metabolic status in RA T cells from those in cancer cells. Excess production of reactive oxygen species and a defect in lipid metabolism characterizes metabolic conditions in SLE T cells. Owing to increased production of the glycosphingolipids lactosylceramide, globotriaosylceramide and monosialotetrahexosylganglioside, SLE T cells change membrane raft formation and fail to phosphorylate pERK, yet hyperproliferate. Borrowing from cancer metabolomics, the metabolic modifications occurring in autoimmune disease are probably heterogeneous and context dependent. Variations of glucose, amino acid and lipid metabolism in different disease states may provide opportunities to develop biomarkers and exploit metabolic pathways as therapeutic targets.

## Introduction

More than 90 years ago, physician-scientist Otto Warburg proposed that cancer is, in principle, a metabolic disease characterized by a mitochondrial defect that shifts energy production towards glycolysis [1]. The so-called Warburg effect has given rise to the concept that abnormal cellular behavior may have its roots in bioenergetics and has nurtured the hopes that metabolic differences between cells offer new targets for low-toxicity therapeutic interventions. Warburg’s discovery has equally encouraged the idea that metabolic intermediates may have diagnostic value, and the almost universal trait of malignant cells massively upregulating glycolysis is exploited in positron emission tomography imaging.

Over the last 90 years, it has become obvious that metabolic switches enable cells to adapt to their bioenergetic and biosynthetic needs, respond to changing requirements for survival, expansion and longevity, and match nutrient availability and functional necessities. Not surprisingly, the need for bioenergetic plasticity is highly relevant for immune cells, which have to abruptly convert from the resting state into battle mode. Bioenergetics are particularly important in autoimmune diseases that are associated with chronic, decade-long immune activation.

Autoimmunity results from abnormal innate and adaptive immune responses that occur in defined tissue sites and often is combined with a systemic inflammatory syndrome. Inflammation is now recognized as a risk factor for inducing insulin resistance and metabolic syndrome [2], maintained by adipose, muscle and hepatic tissues. This review will focus on the metabolic status of individual cells in the immune system, with special emphasis on T lymphocytes as their longevity and memory functions make them critical drivers in autoimmune disease. Here, we summarize what is currently known about metabolic strategies of immune cells in autoimmune disease. The knowledge base about normal and abnormal metabolic adaptations of cells undergoing rapid cellular growth has mostly been built by cancer biologists. Cancer cells and immune cells share commonalities when it comes to ensuring sufficient metabolic flux and bioenergetics for macromolecule synthesis, cell growth and expansion [3]. Detailed studies in cancer bioenergetics have revealed unexpected complexity and context-dependent metabolic switches. Data emerging in human autoimmune disease reveal a similar complexity, with unanticipated metabolic profiles, promising great potential for immunomodulatory therapy via redirecting cellular metabolism.

## Metabolic regulation of normal immune responses

**Figure 1:**
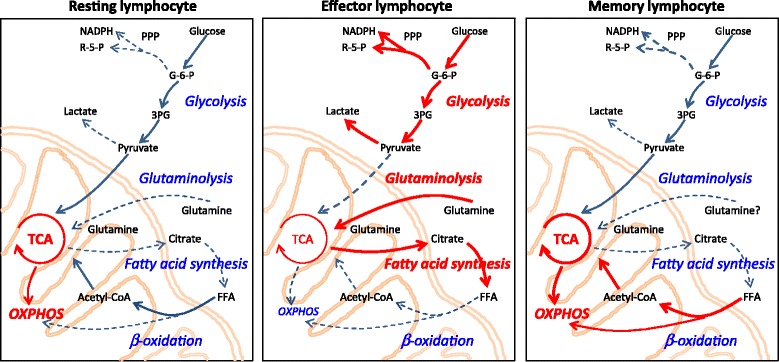
**Metabolic pathways match T cells’ functional demands.** Schematic diagrams of metabolic pathways employed by T cells at different stages of activation and differentiation. Dominant pathways are indicated as red cascades. Blue arrows show pathways that are used at a steady level, and dashed arrows indicate pathways that might be utilized but are insufficiently investigated. (Left) Resting lymphocytes generate energy from glucose, fatty acids and amino acids. Most ATP is produced in mitochondria by fermentation of acetyl-coenzyme A (CoA) in the tricarboxylic acid (TCA) cycle and oxidative phosphorylation (OXPHOS). (Middle) Effector lymphocytes (activated lymphocytes) swiftly and massively upregulate glycolysis and glutaminolysis, while keeping the TCA cycle low. These cells switch lipid metabolism from beta-oxidation towards fatty acid synthesis (lipogenesis). (Right) Memory lymphocytes mainly use beta-oxidation to support their energy needs. 3PG, 3-phosphoglycerate; FFA, free fatty acid; G-6-P, glucose-6-phosphate; NADPH, nicotinamide adenine dinucleotide phosphate; PPP, pentose phosphate pathway; R-5-P, ribose 5-phosphate.

To protect the host from infections and malignancies, immune cells need to respond promptly to antigens and danger signals, including massive expansion of T cells and B cells, migration of cells to relevant tissue sites, and synthesis of cytokines and effector molecules. Accordingly, immune stimulation imposes considerable demands for energy and biosynthetic precursors. Lymphocytes fulfill these demands through swift metabolic changes and rapidly generate energy and building blocks [4,5] (Figure 1). During their life cycle, lymphocytes transition between periods of rest and activity, enforcing great flexibility in metabolic adaptations. Naïve and effector T cells differ greatly in their energy needs and in the means to generate energy [6] (Table 1). Distinct T-cell subsets display unique metabolic programs, and data from metabolomics studies and real-time bioenergetics analyses support the concept that wide variations exist between CD4 and CD8 T cells [7], and between naïve, memory and effector T-cell subpopulations [8,9]. Environmental conditions, such as transitioning from normoxia to hypoxia, may impose additional needs to adapt metabolic programs [10,11]. In essence, each T-cell subset has its very own metabolic profile.

**Table 1:** **Dominant metabolic pathways in resting and activated T cells**

**Metabolic pathway**	**Naïve resting cells**	**Effector cells**	**Memory cells**
Glycolysis	+	+++	+
Pentose phosphate pathway	+	+++	?
Tricarboxylic acid cycle	++	++	+++
Oxidative phosphorylation	++	+	++
Glutaminolysis	+	+++	?
Fatty acid oxidation	++	−/+	++

+, Mild activity; ++, high activity; +++, intensive activity; −/+, context-dependent activity; ?, unknown.

**Table 2:** **Disease-specific metabolic abnormalities in rheumatoid arthritis and systemic lupus erythematosus**

	**Rheumatoid arthritis**	**Systemic lupus erythematosus**
Total oxidative status (serum MDA level)	Low	High
T-cell oxygen consumption	?	High
T-cell TCA cycle activity	?	High
T-cell ROS level	Low	High
T-cell glycolytic activity	Low	?
Serum metabolites	–	Low glycolysis
		Low TCA cycle
		Low fatty acid oxidation
		Low amino acid metabolism
		Low long-chain fatty acids
		High free fatty acids
Synovial fluid metabolites	Low glucose	–
	Low fatty acids	
	High glutamine	
	High amino acids	

MDA, 3,4-methylenedioxyamphetamine; ROS, reactive oxygen species; TCA, tricarboxylic acid.

Pathogenic T-cell populations can be expected to display metabolic and energy signatures. Human autoimmune diseases typically proceed over decades and involve robust memory responses [12]. Disease-relevant T cells depend on long-lasting energy supply. *Vice versa*, the metabolic status of the cell affects its specification and lineage commitment and thus greatly influences the representation of functional effector cells in the host’s immune system.

As an overarching rule, activated effector T cells are anabolic, employing primarily glucose as their carbon source and utilizing glycolysis for fast access to adenosine triphosphate (ATP). Memory cells are catabolic, able to metabolize fatty and amino acids in addition to glucose, and depend on oxidative phosphorylation (OXPHOS) to generate ATP [9] (Table 1). T cells and B cells seem to have evolved distinct approaches to generate energy and macromolecules [13]. Upon stimulation, B cells proportionally increase lactate production and oxygen consumption, optimizing the use of cytoplasmic glycolysis and mitochondrial energy generation. In contrast, T cells tune down their glycolytic flux when resting and disproportionally increase this pathway when encountering antigen. B cells thus thrive in different microenvironments than T cells. T cells effectively utilize glucose via glycolysis, glutamine via glutaminolysis and fatty acid via beta-oxidation, to refill the tricarboxylic acid (TCA) cycle and fuel OXPHOS. We will briefly review the major metabolic pathways to provide the appropriate context to compare the metabolomics of normal and dysfunctional immune responses.

### Glucose and glycolysis

Glucose serves as the primary source for the generation of ATP in the immune system, and is essential for both resting and activated lymphocytes [14] (Figure 1). Nonactivated T cells and B cells predominantly oxidize glucose-derived pyruvate in the TCA cycle and access lipids and amino acids as needed. The TCA cycle generates nicotinamide adenine dinucleotide and reduced flavin adenine dinucleotide used to fuel OXPHOS, an oxygen-dependent process in mitochondria that is highly efficient in producing ATP. The end product of glycolysis, pyruvate, is imported into the mitochondria, decarboxylated to acetyl-coenzyme A (CoA), and then condensed with oxaloacetate to form citrate. Citrate can be exported from the mitochondria via the malate–citrate shuttle system and used as a substrate for ATP citrate lyase. ATP citrate lyase catalyzes the formation of acetyl-CoA and oxaloacetate from cytosolic citrate and CoA in the presence of ATP. Accordingly, ATP citrate lyase serves as a cross-link between glucose and fatty acid metabolism.

Upon recognition of foreign antigen and receipt of appropriate stimulatory signals, T cells become activated and profoundly shift their metabolic program towards aerobic glycolysis for ATP generation, which is less efficient but fast in providing the needed energy. During glycolysis, a molecule of glucose is broken down into two molecules of pyruvate, while yielding two molecules of ATP. Activated T cells convert pyruvate into lactate rather than acetyl-CoA, even in the presence of sufficient oxygen, a process known as aerobic glycolysis or the Warburg effect.

Mechanistically, upregulation of the transcription factor c-Myc is critical in boosting activation-induced glycolysis [15]. c-Myc-dependent transcription directly targets several glycolytic enzymes, but is not essential for fatty acid oxidation and OXPHOS. c-Myc target genes include glucose transporter 1 (*Glut1*), the main glucose transporter in lymphocytes. Glut1 is not expressed at significant levels on the surface of resting T cells, but is rapidly translocated to the plasma membrane through the Akt signaling pathway, which also increases glycolysis by promoting the activities of the rate-limiting glycolytic enzymes hexokinase and phosphofructokinase. Glut1 induction greatly eases the delivery of glucose to T cells, and is considered an essential step in supporting T-cell responsiveness.

Macintyre and colleagues identified *Glut1* as being selectively essential for T-cell activation [16]. *Glut1* deficiency severely impaired T-cell glucose metabolism and decreased effector T-cell differentiation. On the contrary, regulatory T cells were functionally unaffected and able to suppress inflammation regardless of Glut1 expression. Glut1-dependent glycolytic reprogramming has also been implicated in T-cell helper function in antibody production [13].

Although glycolysis provides less ATP than OXPHOS, favoring glycolysis provides T cells with a means of generating the biosynthetic precursors that are required for the synthesis of amino acids, nucleic acids and lipids (Figure 1). Glucose is therefore the optimal energy carrier for T cells and their functionality is closely connected to how they access and break down this carbohydrate. B cells require glucose not only as a source of ATP, but rely on glucose for *de novo* lipogenesis [17]. The reliance on glucose as a supplier of biosynthetic precursors predicts that the level of glycolytic activity might directly influence the ability of activated T cells to become either effector or long-lived memory cells [18]. Memory CD8^+^ T cells possess a markedly increased mitochondrial respiratory capacity when compared with effector T cells [19], implicating OXPHOS as their major energy source.

In essence, T cells depend on glycolysis to support their unique demands for rapid expansion and differentiation into distinct effector populations and have remarkable plasticity to match metabolic and functional activities.

### Glutamine and glutaminolysis

Besides glucose, amino acids are key nutrients for T cells because they can serve both as a fuel source and as a pool of biosynthetic precursors for protein and nucleic acid biosynthesis (Figure 1). T-cell activation imposes acute and delayed demands for protein synthesis. Elegant studies have implicated amino acid transporters as absolute requirements for T cells to adequately respond to antigenic challenge and to undergo clonal expansion and effector differentiation [5]. Specifically, loss of the System L transporter Slc7a5, which mediates uptake of large neutral amino acids, prevents the proliferation and differentiation of CD4^+^ and CD8^+^ T cells, while leaving the ability of CD4^+^ T cells to differentiate into regulatory T cells unaffected. Slc7a5-null T cells fail to increase glutamine and glucose uptake and do not switch to aerobic glycolysis after T-cell receptor stimulation. Cutting the supply of amino acids results in insufficient activation of the amino acid monitor mammalian target of rapamycin complex 1 (mTORC1), which is required for the differentiation of CD4^+^ cells into T-helper (Th)1 and Th17 subsets, while suppressing the differentiation of regulatory FoxP3^+^ T cells [20]. mTORC1 has also been implicated in regulating the differentiation and migratory ability of CD8^+^ cytotoxic T cells [21].

Among the amino acids, glutamine appears to be particularly important. T-cell activation induces a substantial increase in the import of glutamine, but not glutamate [22]. T cells consume glutamine at rates comparable with or even higher than glucose [23]. During glutaminolysis, the amino acid is diverted into metabolic intermediates, such as pyruvate and glutamate. Scientists have long known about the absolute requirement for glutamine in proliferating T cells and have supplemented tissue culture media for T-cell cultures with glutamine.

Recent studies by Nakaya and colleagues have clarified some of the contributions that glutamine makes to T-cell immunity [24]. CD4 T cells uptake glutamine through the ASC amino-acid transporter 2 (ASCT2) and this process influences the development of proinflammatory Th1 and Th17 cells *in vitro* and *in vivo*. Th2 and regulatory T-cell-dependent immune responses are unaffected by the genetic ablation of ASCT2. Activated ASCT2^−/−^ T cells also have reduced glucose uptake, lactate production and oxygen consumption, suggesting that glutamine has a key regulatory role in how T cells respond to abrupt changes in their metabolic needs.

In addition to serving as a basic building block for protein synthesis, glutamine contributes to other processes important for proliferating T cells, including fatty acid synthesis, nucleotide synthesis and redox control. In activated lymphocytes, citrate derived from glycolytic pyruvate is exported out of the mitochondria and used in lipid synthesis. Glutamine-derived α-ketoglutarate contributes to the production of citrate by forward flux through the TCA cycle and malic enzyme-dependent production of pyruvate [25], thus replenishing TCA cycle intermediates that are otherwise extracted for biosynthesis in a process named anapleurosis. Citrate can then be used for the production of acetyl groups for fatty acid synthesis. This pathway allows T cells to utilize glucose-derived citrate to leave the mitochondria. Also, α-ketoglutarate can provide precursors for polyamine synthesis, indispensable for nucleotide synthesis. Finally, glutamate, the first product of glutamine oxidation, serves as a metabolic nexus for the synthesis of glutathione, critically influencing the redox status of lymphocytes.

### Lipid metabolism

The key role of glucose and glutamine in sustaining cell growth, proliferation and effector function of T cells is undebated. Less is known about fatty acid metabolism and how it regulates T-cell fate and function (Figure 1, Table 1). In this context, it is important to consider kinetics of cellular responses, in that glucose and glutamine are rapidly available and are easy to metabolize. Fatty acids may be more important for long-term energy storage. As signaling molecules and membrane building blocks, they play a compulsory role in the cell’s life cycle. Like few other cell types, T cells need to be able to abruptly transition from quiescence to massive expansion. Accordingly, they switch their lipid metabolism from energy generation through fatty acid oxidation to fatty acid biosynthesis for membranes and signaling molecules [26] (Figure 1). During steady state, both naïve and memory T cells catabolize fatty acids through beta-oxidation into acetyl-CoA, which fuels the TCA cycle to provide most of the metabolic support for basic cellular functions [27]. After activation, beta-oxidation is minimized while other metabolic pathways, including glycolysis and glutaminolysis, increase. Lipids, such as phospholipids, glycolipids and cholesterol, are the most abundant molecular species within cell membranes. Lymphocytes are equipped with the enzymatic machinery to utilize acetyl-CoA and build complex fatty acids. Expression of enzymes needed for fatty acid metabolism is markedly upregulated post-stimulation, including the two key rate-limiting enzymes fatty acid synthase and stearoyl-CoA desaturase-1 [26]. T-cell activation is also associated with prompt induction of long-chain acyl-CoA synthetases and lysophosphatidylcholine acyltransferases, known to catalyze the formation of fatty acyl-CoA. Notably, removal of the stimulus in proliferating T cells results in reversal of the lipid metabolism to resting state conditions and the immediate hold of proliferation [28]. Accordingly, CD8 T cells with a defect of *de novo* lipogenesis fail to undergo T-cell expansion, unless they are supplied with exogenous fatty acids [29]. In essence, proliferating lymphocytes appear to draw on external and internal lipid sources to satisfy their enormous need for membrane building blocks.

Lipids integrated into membranes have a major influence on how T cells function. Lipid rafts (also called lipid microdomains), which act as platforms for propagation of signal transduction cascades, are composed primarily of phospholipids, sphingolipids and cholesterol. Phospholipids are rich in unsaturated acyl chains and tend to pack loosely into a liquid-disordered phase. Such membrane domains are considerably more fluid, allowing rapid lateral movement within the lipid bilayer. By contrast, sphingolipids have long and largely saturated acyl chains and easily pack tightly into a bilayer. Adding cholesterol to the acyl chains further stabilizes the membranes. Cholesterol-containing sphingolipid microdomains therefore present as a liquid-ordered phase. Miguel and colleagues have examined the membrane lipid order in T lymphocytes using a fluorescent lipid probe to distinguish liquid-ordered (raft) and liquid-disordered (nonraft) membranes [30]. They found proliferative activity closely correlated with the degree of membrane lipid order. High lipid-order CD4 T cells proliferate robustly to T-cell receptor activation, while intermediate-order cells have moderate proliferative ability and low-order T cells have literally no response. Remarkably, different cytokine-producing cells fall into distinctive membrane lipid-order populations; for example, interferon gamma-producing CD4 T cells accumulate among intermediate lipid-order populations, whereas interleukin (IL)-4-producing CD4 T cells are localized within the high-order populations. Pharmacologic manipulation of membrane order by adding 7-ketocholesterol and cholesterol into the culture media, which has been shown to reduce lipid order, inhibits CD4 T-cell proliferation and IL-2 production.

Lipid metabolism is thus critically important in determining access to stored energy, but even more relevant by altering the composition of cellular membranes.

## Metabolic regulation of pathogenic immune responses

### Rheumatoid arthritis

Rheumatoid arthritis (RA) is a prototypic autoimmune disease, characterized by persistent immune activation [31,32]. The strongest genetic risk factors have been associated with the human leukocyte antigen region and with genes setting cytoplasmic signaling thresholds [33]. Pathogenic immune functions include excess cytokine production, dysregulated proliferation of synovial fibroblasts, formation of complex lymphoid microstructures in inflamed joints, autoantibody production, and uncontrolled activity of bone-destructive osteoclasts. The prevailing concept has been that identifying the autoantigens, assumed to be the original trigger, would reveal the pathogenesis. Over the last decade, antigen-nonspecific abnormalities have been implicated in the dysregulated immune system of RA patients and the question arises of to what extent metabolic dysregulation contributes to the breakdown of self-tolerance. Indeed, several glycolytic enzymes, including glucose-6-phosphate isomerase, aldolase and enolase, have been identified as antigens recognized by autoantibodies [34-36]. This may reflect the propensity of RA patients to break self-tolerance against a wide variety of antigens. How autoantibodies to glycolytic enzymes would alter metabolic competence of immune cells is unclear. Proteomic analysis of synovial fluid has revealed that proteins involved in glycolytic pathways are highly expressed in RA patients, but not in synovial fluids from osteoarthritis patients, which is in accordance with upregulation of glycolytic flux in synovial lesions [37].

**Figure 2:**
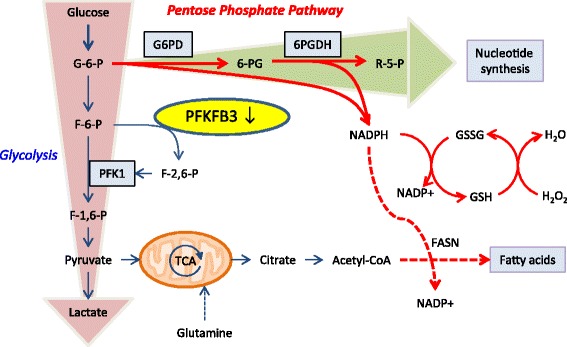
**Metabolic reprogramming in rheumatoid arthritis T cells.** In contrast to healthy CD4 T cells, rheumatoid arthritis T cells fail to upregulate glycolytic activity due to the insufficient induction of 6-phosphofructo-2-kinase/fructose-2,6-bisphosphatase 3 (PFKFB3), a key regulatory enzyme in the glycolytic pathway. Deficient activity of PFKFB3 shunts glucose towards the pentose phosphate pathway and increases intracellular NADPH levels, hence unbalancing the cell’s redox status. 6PGDH, 6-phosphogluconate dehydrogenase; F-1,6-P, fructose-1,6-bisphosphatase; F-2,6-P, fructose-2,6-bisphosphatase; F-6-P, fructose-6-phosphate; FASN, fatty acid synthase; G-6-P, glucose-6-phosphate; G6PD, glucose-6-phosphate dehydrogenase; GSH, glutathione; GSSG, glutathione disulfide; NADPH, nicotinamide adenine dinucleotide phosphate; PFK, 6-phosphofructo-2-kinase; R-5-P, ribose 5-phosphate; TCA, tricarboxylic acid.

A recent study has examined the metabolic status of CD4 T cells in RA patients (Figure 2) [38]. The analysis focused on naïve CD4 T cells, thus excluding T cells directly involved in the inflammatory process itself. When stimulated through the T-cell receptor and transitioning into effector T cells, such naïve CD4 T cells are expected to swiftly upregulate aerobic glycolysis, following a classical Warburg effect. Remarkably, RA T cells failed to produce as much ATP and lactate as healthy control T cells, while vigorously proliferating [38]. Molecular analysis of the underlying defect identified the enzyme 6-phosphofructo-2-kinase/fructose-2,6-bisphosphatase 3 (PFKFB3) [38]. PFKFB3 is a rate-limiting enzyme in the glycolytic pathway, making it an ideal target for regulatory interference. PFKFB3 is a bifunctional enzyme that prompts glycolytic flux by generating fructose-2,6-bisphosphate, an allosteric activator of the key glycolytic enzyme 6-phosphofructo-1-kinase. PFKFB3 is considered to have a high ratio of kinase to phosphatase activity and converts fructose-2,6-bisphosphate to fructose-6-phosphate when functioning as a phosphatase. The study employed a gene expression screen for 29 glycolysis-related markers, and PFKFB3 was the only marker that was significantly suppressed in RA T cells.

The defect in glycolysis has consequences for the affected T cells (Figure 2). Not only do RA T cells produce less ATP and lactate, they also shunt glucose towards the pentose phosphate pathway, and generate increased levels of nicotinamide adenine dinucleotide phosphate (NADPH), the principal intracellular reductant [38]. NADPH converts glutathione disulfide to its reduced form glutathione, eventually diminishing intracellular reactive oxygen species (ROS). ROS have traditionally attracted attention for their potential to directly harm proteins, lipids, DNA, cellular organelles and membranes. Recently, ROS have been recognized as important regulators of intracellular signaling pathways. Previous studies have connected increasing risk for arthritic disease with NOX2 deficiency. Also, reduced ROS production is associated with increased severity of joint inflammation [39-41]. This indicates a role for oxidative burst in protection from arthritis.

Metabolic consequences of PFKFB3 deficiency in RA T cells are not limited to enhancing NADPH and pentose production. PFKFB3 also represses the activity of autophagy, which is a catabolic process and is upregulated to degrade cytoplasmic contents under energy deprivation [42]. Considering their decreased glycolytic flux, RA T cells would be expected to resort to enhanced autophagic activity to fulfill their demands for energy and biosynthetic macromolecules. However, RA T cells are unable to upregulate autophagic flux and are forced into apoptosis in the presence of the autophagy inhibitor 3-methylamphetamine [38]. This insufficient autophagic activity in RA T cells can be, at least partially, repaired by overexpression of PFKFB3, which suggests an important role of PFKFB3 in the coordination of the autophagy machinery.

Why RA T cells fail to induce PFKFB3 and essentially commit to an anti-Warburg effect is not understood. However, this is not the first abnormality in the naïve CD4 T-cell pool of RA patients. Over the last decade, it has become obvious that T cells in RA patients are prematurely aged [43-46]. The accelerated aging phenotype of RA T cells includes shortening of telomeres, loss of CD28 and reduced efficiency of DNA repair mechanisms [46-49]. T-cell aging has been associated with resetting of signaling thresholds due to age-related changes in phosphatase activity [50,51]. It is currently unknown whether the metabolic reprogramming of RA T cells is mechanistically connected to the pre-senescent phenotype of the cells. It is conceivable that the energy deficiency of the cells shortens their lifespan, thus imposing proliferative pressure that ages the T-cell compartment. Alternatively, senescence-associated shifts in gene expression could affect production of glycolytic enzymes and thus result in altered glycolytic flux. Independent of whether glycolytic insufficiency precedes or follows the process of T-cell aging, lower ability to generate ATP should render T cells sensitive to apoptosis and thus cause lymphopenia-induced T-cell turnover. Lymphopenic hosts are more likely to have autoreactive T cells, because homeostatic T-cell expansion relies on recognition of autoantigens [32].

### Systemic lupus erythematosus

The wide range of autoantibodies in systemic lupus erythematosus (SLE) has fostered concepts of intrinsic B-cell abnormalities in this autoimmune disease [52]. Convincing data have, however, revealed that T cells critically participate in the pathogenesis of SLE due to their capabilities to guide B cells in autoantibody production. Both abnormal T-cell activation and signaling are suspected to contribute to aberrant B-cell response. Efforts to understand how dysfunctional T cells promote disease processes in SLE have recently focused on cell-intrinsic abnormalities, including metabolic shifts in T cells from SLE patients.

In contrast to healthy lymphocytes, lupus T cells secure ATP production through OXPHOS, rather than upregulating aerobic glycolysis [53]. Splenocytes from lupus mice have been reported to increase glucose oxidation by 40% due to enhanced activity of the TCA cycle activity. Glycolytic activity in chronically stimulated human T cells can be significantly lower than in acutely activated cells [53]. Underlying mechanisms are unknown, but it has been speculated that reduced CD28 expression may go hand in hand with less active aerobic glycolysis. SLE T cells have elevated mitochondrial membrane potential, produce more ROS and have reduced intracellular glutathione [54,55], possibly caused by the acceleration of the TCA cycle resulting in excessive ROS generation due to the leakiness of the electron transport chain. Convincing evidence has accumulated over the last decade that SLE is a disease associated with increased oxidative stress [56] and excessive oxidative capacity has been implicated in underlying immune dysfunction, autoantibody production and in the cardiovascular complications of the disease. Evidence has been provided that dysfunctional mitochondria are the main source of excess ROS in SLE [57].

A study by Kato and Perl linked IL-4 and IL-17 production in lupus T cells with increased activity of mTORC1 [58]. Excessive ROS production and increased mTORC1 activity have prompted clinical trials designed to correct these metabolic defects, ranging from inhibition of mTORC1 by rapamycin to reversal of glutathione depletion by *N-*acetylcysteine [59,60]. The kinase activity of mTORC1 is under regulatory control of the cell’s major energy sensor 5' adenosine monophosphate-activated protein kinase (AMPK). Spontaneous mTORC1 activity would suggest that AMPK is insufficiently activated in SLE T cells, which is unexpected under conditions of highly activated mitochondrial activity and ROS release. A metabolomic analysis of SLE sera has revealed that energy biogenesis from all sources is diminished. Based on a broad analysis of metabolites, glycolysis, fatty acid beta-oxidation and amino acid metabolism all appear to be dampened, while levels of free fatty acids are increased, supporting the notion that SLE is associated with abnormalities in lipid metabolism [61]. Diminished energy biosynthesis should activate AMPK and lead to subsequent downregulation of mTORC1. Further studies are urgently needed to integrate these findings and to connect them to the pathogenic role of lymphocytes in the disease.

**Figure 3:**
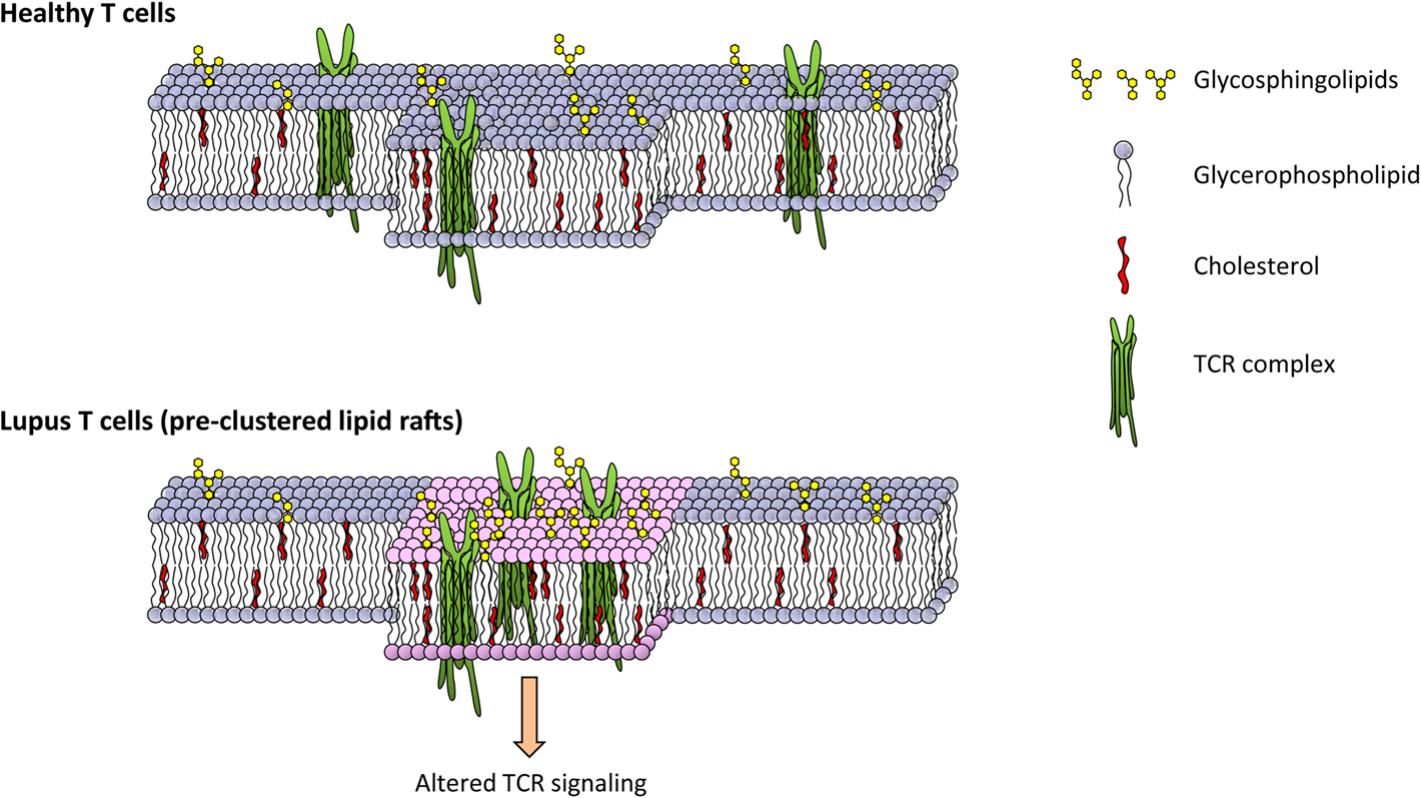
**Altered membrane lipids in lupus T cells.** The amount of glycerophospholipid, glycosphingolipids and cholesterol is tightly regulated and critical for T-cell receptor (TCR) signaling in healthy T cells. T cells from systemic lupus erythematosus patients exhibit excessive glycosphingolipid homeostasis, leading to aggregated lipid rafting and altered TCR signaling.

In a recent study, McDonald and colleagues investigated the complex crosstalk between lipid metabolism and T-cell dysfunctions in lupus. Compared with healthy controls, CD4 T cells from SLE patients had significantly elevated lipid raft-associated glycosphingolipids [62] (Figure 3). Also, such T cells had elevated expression of Liver X receptor, a member of the nuclear receptor family of transcription factors that function as important regulators of cholesterol and fatty acid homeostasis. Altered glycosphingolipids and cholesterol homeostasis in lipid rafts led to abnormal T-cell receptor signaling, most probably by promoting formation of raft structures and increasing lipid raft localization of critical signaling mediators, such as the protein tyrosine kinase LCK and CD45. Inhibition of glycosphingolipids metabolism normalized CD4 T-cell signaling and decreased anti-double-stranded DNA antibody production by autologous B cells. These data support the notion that lipid biosynthesis is closely correlated with membrane function and setting the threshold for signaling. The molecular mechanisms that drive lipid metabolic dysfunction in T cells in SLE have not been clarified.

### Multiple sclerosis

While not a rheumatic disease, studies on pathogenic pathways in the autoimmune disease multiple sclerosis have been highly informative in deciphering immune abnormalities that lead to immune-driven tissue damage. In terms of metabolic abnormalities, elevated levels of both glutamine and glutamate have been reported in clinical cases of multiple sclerosis [63] and glutamate concentrations have been related to multiple sclerosis severity [64], raising the interesting question of whether the neurotransmitter glutamate could fuel tissue-injurious immunity. The level of glutamate is closely interconnected with glutamine through the glutamate/gamma-aminobutyric acid–glutamine cycle. Besides its role as a neurotransmitter, glutamate is a key source of energy in neurons, glia and immune cells. Lymphocytes possess glutamine synthetase activity, enabling them to synthesize glutamine from glutamate [65]. Following activation, T cells boost glutamine uptake by 5-fold to 10-fold compared with the resting state. Glutamine uptake depends on the transporter ASCT2, a molecule that has recently been implicated in affecting the development of CD4 Th1 and Th17 effector cells via regulating the activity of the kinase mammalian target of rapamycin [24]. Mice deficient for the amino acid transporter ASCT2 are refractory to the induction of experimental allergic encephalomyelitis, an animal model of multiple sclerosis [24].

In essence, T cells depend on transporter-supported glutamine import to nurture their activation and their pathogenic role in central nervous system inflammation.

## Conclusions

Highly proliferative immune cells share with cancer cells the switch to progrowth glycolysis, which secures both ATP and macromolecules. Another key nutrient source is amino acids, particularly the nonessential amino acid glutamine, which provide energy as well as biosynthetic precursors for proteins, nucleic acids and lipids. More needs to be learned about lipid metabolism on the cellular level, because lipids serve as densely-packed energy reservoirs and are essential building blocks for membranes and signaling molecules.

A simple paradigm would assume that chronic autoimmune diseases, which depend on long-lived and highly-differentiated lymphocytes, are a high energy-consuming state susceptible to metabolic manipulations. However, emerging data in RA and SLE attest to the complexity of metabolic programs in chronic autoimmunity. RA T cells have a defect in PFKFB3, a gatekeeper enzyme in the glycolytic pathway, leaving them energy deprived. Their energy deprivation is sufficient to redirect glucose utilization and affect the cells’ redox status, rendering them apoptosis sensitive and ROS depleted. Conversely, lupus T cells appear metabolically more active, producing excess ROS. Signaling abnormalities in lupus T cells are associated with alterations in the lipid composition of cell membranes. Differences in the redox status of RA and SLE patients, with oxidative pressure in SLE and reductive pressure in RA, suggest fundamentally distinct metabolic programs in both disease processes, which may reflect differences in how nutrients are handled in different microenvironments or may indicate differences regarding the metabolic niches to which lymphocytes are exposed.

Data from RA and SLE challenge the simplified model that surplus immune activation is equivalent to surplus nutrient supply and instead give rise to the concept that disease-specific patterning of metabolic abnormalities may exist. Disease-specific abnormalities have implications for diagnostic and therapeutic approaches, because a one-size-fits-all approach may not work. However, modifying cell-internal metabolism in T cells represents a novel therapeutic opportunity to treat autoimmunity. This would indeed be good news for rheumatologists because it may pave the way to highly sophisticated disease-adapted immunomodulation instead of using broad-based, nonspecific immunosuppression.

## Abbreviations

AMPK: 5′ adenosine monophosphate-activated protein kinase
ASCT2: ASC amino-acid transporter 2
ATP: Adenosine triphosphate
CoA: Coenzyme A
*Glut1*: Glucose transporter 1
IL: Interleukin
mTORC1: Mammalian target of rapamycin complex 1
NADPH: Nicotinamide adenine dinucleotide phosphate
OXPHOS: Oxidative phosphorylation
PFKFB3: 6-phosphofructo-2-kinase/fructose-2,6-bisphosphatase 3
RA: Rheumatoid arthritis
ROS: Reactive oxygen species
SLE: Systemic lupus erythematosus
TCA: Tricarboxylic acid
Th: T-helper
